# Supplementary material for: Are carbon emissions trading and green financial instruments synergistic? -Comprehensive quantitative research based on content analysis
Source: PLoS One. 2024 Mar 7;19(3):e0298601. doi: 10.1371/journal.pone.0298601 (PMC10919601; doi:10.1371/journal.pone.0298601)
Supplement: S1 Data — (ZIP) [file pone.0298601.s001.zip › Data/地级市Z_F工作报告.zip!/╔╜╬≈/│ñ╓╬╩╨/└┤╫╘╣1⁄2╓┌║┼í╛┬φ┐╦╩2╛▌═°í┐_2019.docx]

各位代表：

　　现在，我代表市人民政府向大会报告工作，请予审议，并请市政协委员和其他列席人员提出意见。

　　一、2018年工作回顾

　　过去一年，是长治发展进程中意义非凡的一年。一年来，我们坚持以习近平新时代中国特色社会主义思想为指引，在市委的坚强领导下，全面贯彻落实党中央、国务院和省委、省政府各项决策部署，攻坚克难，砥砺奋进，各项工作取得明显成效，各项事业取得显著进步，开创了新时代长治转型发展的新局面。

　　2018年，全市地区生产总值完成1646亿元、增长7.4%，增速高于全国、全省平均水平，总量新增近170亿元，经济总量稳居全省第二位。一般公共预算收入完成150.7亿元，创历史新高。圆满完成了全省考核地市的10类22项区域经济转型指标，排名全省第二。

　　一年来，主要做了以下几个方面的工作：

　　（一）加速推进转型发展。我们坚持把项目建设作为转型发展的切入点和突破口，重点推进了478个5000万元以上的产业转型项目，潞安180、潞宝己内酰胺、全球首条量产化深紫外LED生产线等123个产业转型项目建成投产，全年固定资产投资完成679.6亿元、增长10.7%，转型项目完成投资441.7亿元，占固投总量的65%，高于全省平均水平2.9个百分点；出台了招商引资奖励办法，引进落地378个项目，招商引资项目签约个数、开工个数、开工率均居全省第一，新开工项目到位资金全省第二。我们切实提升传统产业，克服困难，退出煤炭产能195万吨、钢铁产能155万吨，化解焦化产能160万吨，完成“十三五”去产能任务，煤炭先进产能占比达到66%，17座4.3米焦炉全部备案为6米以上大焦炉。我们大力发展新兴产业，装备制造、精细化工、新能源、信息技术等产业保持两位数增长，成功新能源汽车在全国同类型产品中产销量名列前茅，太阳能电池产量达到150万千瓦、增长68.7%。我们全面加力支持企业发展，深入开展入企服务，帮助企业解决难题，全年新增贷款232亿元，为企业减免各类税费40亿元以上；出台支持民营经济发展30条配套措施，筹资9.6亿元盘活金泽玉米公司，新增“小升规”企业43户、“专精特新”企业20户、小微企业5508户，均超额完成省下达目标任务，民营经济增加值完成738.8亿元、增长12.6%。全市煤炭产业增加值占比降低2.8个百分点，高于全省平均水平0.3个百分点，制造业、战略性新兴产业增加值占比分别达到25.6%和6.9%，提高3.3和2.2个百分点，工业结构“反转”迈出坚实步伐。服务业增加值完成690亿元、增长8%，高于GDP增速0.6个百分点。转型发展呈现稳中有进、进中趋优的良好态势，老工业基地调整改造工作受到国务院通报表扬，产业转型升级示范区建设考核全国“优秀”。

　　（二）稳步实施乡村振兴战略。我们坚持把乡村振兴战略作为新时代“三农”工作总抓手，大力发展现代农业，调减玉米种植面积9.7万亩，农产品加工销售收入完成247.5亿元、增长10.8%。在全省率先启动绿色有机旱作农业示范市创建工作，高标准建设了12个绿色有机旱作农业封闭示范区，全省有机旱作农业现场推进会在我市召开。我市被中国粮食行业协会授予“中国小米之都”，新认证“三品一标”农产品183个，“沁州黄小米”入选第二批中国特色农产品优势区，成功举办首届中国农民丰收节分会场活动。全面完成农村承包地确权登记颁证工作，全面推开农村集体产权制度改革。改善535个村25万农村人口安全饮水条件，完成1509公里“四好农村路”建设，改造1.3万户农村危房。我们坚持把脱贫攻坚作为头等大事和第一民生工程，咬定目标，尽锐出战，全力攻坚深度贫困，市本级财政投入扶贫资金2.2亿元、增长35%，产业、生态、教育、健康等各项扶贫措施精准落地，创立消费扶贫“长治模式”，“十三五”易地扶贫搬迁建设任务全面完成，沁源县顺利摘帽，武乡县、沁县进入脱贫摘帽程序，又有302个贫困村、6.49万贫困人口稳定脱贫，贫困发生率由上年的3.3%降至1.1%，脱贫攻坚实现连战连胜。

　　（三）大力推进城市建设。我们围绕省域副中心城市建设，完成了“1+3”行政区划调整，形成了4个市辖区的发展大格局，实现了我市行政区划调整的历史性突破。高起点完成了城市总体规划调整方案、多规合一和滨湖区、高铁片区等重点片区规划编制工作，启动了滨湖区建设。积极争取将总投资55亿元的207、208、309国道市区改线工程改为收费公路，降低了财政负担，309线已改造完工正式通车；襄垣城际连接线、保宁门街主干道全面通车，紫金西街、五针街铁路立交桥主桥投入使用，主城区新建改造城市道路83公里、市政管网887公里。职教园区、长治海关大楼主体基本完工。开工建设了总投资7亿元的生活垃圾焚烧发电厂和污泥餐厨垃圾处置项目，在79个单位开展了生活垃圾分类试点。推进实施天晚集、长丰片区和10个城中村改造，新开工棚户区改造4710套、开工率102.9%，基本建成6552套、建成率131%。长子门小学、火炬中学等3所学校竣工投入使用。各县区围绕“1+6”城镇群建设，同步实施了一大批城建重点工程，“1+6”城镇群的辐射带动能力进一步增强。全市常住人口城镇化率达到53.9%，较上年提高1个百分点。

　　（四）强力推进污染防治。我们坚定把当好首都环保“护城河”作为重大政治使命，全面打响蓝天、碧水、净土保卫战，在全省率先开展道路扬尘和汽车尾气专项整治，完成冬季清洁取暖改造13.9万户，淘汰燃煤锅炉1212台，新增集中供热面积1265万平方米。与山西大学合作成立了长治市固废综合利用研发中心，全年利用煤矸石、粉煤灰等各类工业固废821万吨。完成全市农用地土壤污染状况调查。主城区PM2.5浓度下降10%，空气优良天数增加18天，环境空气质量综合指数全省第三，改善率进入全国169个重点城市前20名，获得环境空气质量改善省级奖励资金4106万元，位居全省第一。主要河流水质优良率全省第一。

　　（五）全力实施创新驱动战略。我们强化创新、注重人才，设立1亿元科技成果转化引导基金和人才资金，成立中科半导体技术研究院、长治大健康产业研究院、海外华人高新技术协会长治工作站等一大批产学研合作平台，与98家企业和科研院所签订合作协议，达成合作事项106项。在全省率先出台人才落户实施细则、人才安居工作实施办法，引进461名硕士研究生以上高素质人才，为704名各类人才提供“拎包入住”的安居住房，成功举办首届长治技能大赛。一年来，新认定高新技术企业38家，完成技术合同交易额19亿元；有效发明专利拥有量689件，万人有效发明专利拥有量两件，均位居全省前列；新培育两个国家、省级“双创”示范基地，7个省级“双创”平台，“双创”工作受到国务院通报表扬，获得中国产学研合作创新与促进奖。

　　（六）不断深化改革扩大开放。我们坚持改革不能落后、改革必须先行，坚定以改促转，不断增强发展的内生动力。开发区改革创新发展成效明显，潞城、沁县、上党、沁源4个开发区成为省级开发区，省级以上开发区达到9个，新批数量全省最多，全市开发区新开工项目112个，开发区投资、产出、税收强度均大幅提升。国企国资改革步伐加快，全面完成驻地17户央企、18户省属企业的10.1万户职工家属区“三供一业”分离移交工作。金融体制改革取得实效，沁源、沁县农信社改制为农商行，在全省率先全面完成农信社改制。对外开放不断扩大，对外通道建设全面提速，太焦高铁顺利推进，长临高速全面通车；开通了长治至晋城、郑州、安阳等城际旅游公交，实行节假日免费、工作日低票价政策，运送游客80万人次，全市旅游人数、旅游收入均保持25%以上的增长；新增振东医药、长清生物等18家进出口实绩企业，进出口总额增长42%，成功集团在巴西建立首家散装工厂，实现了我市企业海外发展的新突破。

　　（七）不断增进民生福祉。我们坚持以人民为中心的发展思想，全力解决好人民群众关心的热点难点问题。全年民生事业支出占到财政总支出的82.9%。省政府“六件民生实事”全面完成。围绕千家万户高度关注的教育问题，加大教育投入、整合教育资源、提升教学质量，全市高考升学率明显提高，二本以上达线人数1.1万人、增长10%，清华北大达线人数19人、增长70%，6个县区全部通过国务院义务教育发展基本均衡评估验收。完成各类职业技能培训5.7万人，城镇新增就业5.3万人，农村劳动力转移就业4.3万人，城镇登记失业率1.9%，低于省控目标2.3个百分点。县乡医疗卫生机构一体化改革基本完成，市二院、市中医院、市中研所附属医院晋升为“三甲”，“三甲”医院达到8所，全省第二。城乡居民基础养老金、基本医疗保险财政补助标准分别提高276元、40元，实现基本医疗保险跨省异地就医直接结算，各项社会保险参保人数创历史新高。城乡居民人均可支配收入分别增长6.5%、8.8%，农村居民收入增幅继续高于城镇居民。成功举办第十届中国曲艺牡丹奖全国曲艺大赛。深入开展扫黑除恶专项斗争，积极创建全国禁毒工作示范城市，全市各类安全生产事故起数和死亡人数分别下降7.7%、5.7%，人民群众的安全感和满意度进一步提高。

　　一年来，我们全面加强政府自身建设，严格落实政府系统全面从严治党主体责任，深入推进党风廉政建设和反腐败斗争，切实加强审计监督，发挥督查利剑作用。自觉接受市人大法律监督、市政协民主监督，办理人大代表建议217件、政协提案332件，办复率100%。我们围绕打造“六最”营商环境，持续深化“放管服效”改革，将企业开办时间压缩至3天，新增市场主体2万户，全市市场主体达到19.5万户、增长11.5%。我们全面改进作风，旗帜鲜明地鼓励干部担当作为、干事创业，通过竞争性评审答辩，积极争取成为冬季清洁取暖试点城市和国家黑臭水体治理示范城市，分别获得16.5亿元、6亿元中央财政奖补资金；全年共争取各类上级资金45亿元，位列全省第一，获得省财政厅2600万元奖励；对所有政府类投资项目进行严格的财政评审，核减投资14.4亿元；全年市本级“三公”经费下降10.8%。我们突破阻力、排除干扰，在主城区开展了“两违”专项整治，拆除违建11万平方米，补缴各项税费15亿元，完善各类审批手续387个，城市用地、建设行为得到有效规范；收回判决七年之久未能执行的阳泉涉黑案件资产，价值2亿元，彰显了法律公平，维护了人民利益。

　　各位代表，成绩来之不易。这是市委正确领导，市人大、市政协有效监督，全市人民凝心聚力、奋勇拼搏的结果。在此，我代表市人民政府，向全市人民，向各民主党派、工商联、无党派人士和各人民团体，向各位人大代表、政协委员，向驻市部队、武警官兵、公安干警，向所有关心支持长治改革发展的各界朋友，表示崇高的敬意和衷心的感谢！

　　在肯定成绩的同时，我们也清醒看到，我市经济社会发展还存在不少困难和问题。突出表现在：一是经济发展水平不高，全市GDP总量尽管在全省排第二，但仅比排在第三的运城高100多亿元，与周边省份的邯郸等地市都有较大差距。二是转型发展任务艰巨，新兴产业正处在逐步成长壮大阶段，“一煤独大”的结构性矛盾尚未根本性改变，服务业发展低于全国、全省平均水平，科技创新能力不强，引领转型的高端人才严重短缺，发展新动能仍然不足。三是社会事业欠账较多，重大交通基础设施建设不足，城乡居民持续增收制约因素较多，决胜脱贫攻坚压力较大，生态环境形势依然严峻，教育发展处于全省中下游水平，医疗、养老等公共服务还不能满足人民群众需要，各领域依然面临许多风险和挑战。四是营商环境优化不够，行政办事效率、便民服务措施还有差距，一些干部思想解放不够、创新意识不强、担当精神不足，形式主义、官僚主义依然存在。对此，我们一定高度重视、切实解决，努力把各项工作做得更好。

　　二、2019年工作安排

　　2019年是新中国成立70周年，是全面建成小康社会的关键之年。今年政府工作的总体要求是：高举习近平新时代中国特色社会主义思想伟大旗帜，坚持新发展理念，聚焦“三大目标”，突出转型项目，实施创新驱动，深化改革开放，提速城市建设，推进乡村振兴，继续打好三大攻坚战，切实防范化解重大风险，加快打造山西重要增长极、建设省域副中心城市、建设美丽幸福长治，以优异成绩迎接中华人民共和国成立70周年。

　　主要预期指标是：地区生产总值增长6.5%左右，固定资产投资增长7%，社会消费品零售总额增长7.5%，一般公共预算收入增长7%，城乡居民人均可支配收入分别增长6.5%和6.5%以上；居民消费价格涨幅控制在3%左右，城镇新增就业4.1万人，城镇调查失业率、城镇登记失业率分别控制在6.5%、4.2%以内。

　　约束性指标是：万元地区生产总值能耗下降3.2%，万元地区生产总值二氧化碳排放量下降3.9%，万元地区生产总值用水量下降2.4%。环境质量改善指标和主要污染物总量减排指标，完成省下达年度目标任务。

　　各位代表，实现上述目标，任务艰巨、责任重大。我们一定扛起长治改革发展稳定的重任，准确把握重大政策取向，抢抓重大战略机遇，按照“巩固、增强、提升、畅通”八字方针，改革创新、奋发有为，推动长治高质量发展取得更大突破。今年重点抓好九个方面的工作。

　　（一）着力转型项目建设，增强高质量发展后劲。转型项目建设始终是资源型地区加速转型发展的关键所在。我们将坚决按照省委、省政府深化转型项目建设年的要求，加快改造提升传统产业，加速培育新兴产业集群，构建长治现代化经济体系，重点实施315项产业转型项目，完成投资490亿元，以项目建设带动产业转型升级，推动高质量发展。

　　培育新兴产业集群。聚焦制造业发展，以高端化、绿色化、智能化、融合化为方向，重点培育八大优势产业集群：现代煤化工产业集群，依托潞安180项目，进一步延伸上下游产业链条，重点推进潞安煤制油180万吨产能扩产至240万吨、20万吨高密度航空煤油及柴油等12个项目，完成投资30亿元。依托潞宝己内酰胺产业链条延伸，重点推进10万吨聚合切片、6万吨短纤维及3万吨石蜡等10个项目，完成投资26亿元。国家级半导体光电产业集群，依托中科半导体技术研究院，重点推进中科潞安深紫外LED芯片二期、衬底材料、外延、封装、应用等8个项目，新上高科LED外延芯片、白光LED封装、家电产品配套等6个项目，完成投资25亿元。新能源汽车产业集群，支持成功集团建设国家重点实验室，抓好物流电动汽车研发及充换电设施建设项目，积极引进氢燃料电池，完成投资8亿元。医药健康产业集群，依托康宝集团、振东集团、长治大健康产业研究院，重点推进康宝血液制品、振东抗肿瘤药物、太行药业植物干细胞、大健康化妆品、食品等11个项目，完成投资18亿元。军民融合产业集群，依托军民融合示范基地，积极创建军民融合示范市，重点推进淮海军民融合科技产业园和平顺航天工业园二期建设，完成投资7亿元。通用航空产业集群，依托临空产业园和通用产业园建设，重点推进星箭察打一体无人机生产线、丰荣航空维修等6个项目，完成投资8亿元。信息技术产业集群，依托智慧长治建设，重点推进创盈智能终端产业园、遥感卫星地面接收站网、大数据中心等6个项目，完成投资12亿元。固废综合利用产业集群，依托长治市固废综合利用研发中心，抓住国家建设100个大宗固废综合利用基地的机遇，建立固废利用循环经济模式，全年利用工业固废800万吨以上。

　　改造提升传统产业。以能源革命为突破，加大传统产业技改力度，重点推进93个工业转型升级和工业技改项目，完成投资146亿元。“稳煤”，建成投产3座矿井，新增产能1040万吨，增加先进产能供给，力争先进产能占比达到70%以上，全年煤炭产量稳定在1.1亿吨左右。“聚焦”，围绕焦化产业整合集约发展，将全市1700万吨产能全部集聚到9大焦化项目。“提冶”，鼓励首钢长钢扩大多元化产品规模，支持长信公司生产带钢等新产品，确保全年钢铁产量达到570万吨、增长10%。“输电”，推进潞光电厂2×66万千瓦项目10月份正式投产，确保漳泽电厂2×100万千瓦改扩建项目复工，力争赵庄电厂2×66万千瓦项目复工，开工以上3个电厂电力外送通道建设，完成投资50亿元。“上新”，支持武乡、长子区块煤层气开采，新增煤层气开采量3亿立方米，做好应急储气能力建设。推进太阳能、风能、生物质能项目建设，确保500兆瓦国家光伏技术领跑基地6月底前并网发电，建设长治光伏发电技术创新国家重点实验室和光伏创新技术的试验、中试基地，加快壶关华能风电、巨鹏生物质燃料乙醇项目建设。

　　大力发展现代服务业。加快构建立体式现代物流格局，加速“公转铁”运输结构调整，依托现有的瓦日、太焦、长邯等铁路运输通道，在潞城、壶关等5个县区建设大型物流基地，完成投资16亿元。加快发展现代商贸业，在4个新设市辖区分别规划布局1个大型商业综合体。大力发展电子商务，新培育1个省级示范基地、1个农村示范县。持续扩大消费需求，推进工业品下乡、农产品进城，搭建更为完善的双向商贸流通网络，大力培育消费新业态、新热点，推动消费提质升级。力争全市服务业增加值占地区生产总值比重再提高1个百分点。

　　（二）着力创新驱动，培育高质量发展新动能。实施创新驱动发展战略是实现高质量发展的重大举措。我们将强化科技引领，发挥人才作用，提升质量水平，加快新旧动能接续转换，实现长治“制造”向长治“创造”的飞跃。

　　深化创新驱动。充分发挥长治市工业技术研究院、长治能源革命研究院等创新平台的作用，加大科研投入，至少完成5项重大科技成果转化，完成技术合同交易额15亿元以上，新增15家高新技术企业。加强知识产权保护，确保完成发明专利申请300件，万人有效发明专利拥有量达到2.1件。推动现有10个国家、省级“双创”示范基地提档升级，重点推进“双创”梦工厂、唯美诺二期工程建设，提高“双创”科技含量，提升“双创”基地集聚力，打造长治“双创”升级版。

　　突出人才支撑。实施“潞商潞才回乡创业创新工程”，继续落实人才政策，发挥海外华人高新技术协会长治工作站桥梁纽带作用，建立驻京、驻粤及海外人才工作站，力争引进500名硕士研究生以上高素质人才、百名海归人才，签约10个创新项目。统筹推进实用人才、乡土人才和熟练产业工人的培养，办好第二届长治技能大赛、“五小”竞赛、青年创新创业大赛，加快推进高技能人才培训基地、技能大师工作室建设，培育一批独具特色的长治工匠。

　　提升质量标准。大力开展国家标准化综合改革试点，制定10个新兴产业和传统产业技改领域技术标准，培育3个高新技术产业、5个服务业国家标准化试点示范项目、10个消费品标准化示范单位。积极争创全国质量强市示范市，加快推进国家煤基合成油质检中心建设，支持企业提升产品质量，鼓励企业走出去推广品牌，力争推出10家山西省名牌企业、2个地理标志保护产品。

　　（三）着力改革开放，激发高质量发展活力。改革不停顿、开放不止步。我们将以改革开放永远在路上的奋斗姿态，大力弘扬敢闯敢试、敢为人先的精神，持之以恒、久久为功，努力形成更高层次改革开放新格局，走在全省改革开放“第一方阵”。

　　推进开发区改革创新发展。加大开发区投资、产出和税收强度的考核力度，开展土地利用节约集约评价，进一步完善基础设施，建成标准化厂房30万平方米，收储土地5000亩，提高开发区项目承载能力，高新区、经开区至少新上5个10亿元以上产业转型项目，其他开发区至少新开工2个10亿元以上新兴产业项目。持续深化“三化三制”改革，推动潞城经开区民营企业化管理改革试点取得实质性突破，真正把我市开发区的数量优势转化为质量优势。

　　深化国企国资改革。继续化解国企历史包袱，巩固企业办社会分离移交成果，积极推动企业办市政社区等管理职能移交工作。全力做好处僵治困工作，推动市场化出清，扎实做好市属国有困难企业代缴基本养老、基本医疗保险工作，对下岗职工集中托管，对困难企业资产进行集中经营，盘活闲置资产，实现国有资产保值增值。稳步推进混合所有制改革，完成国信担保等3家企业改革试点工作。

　　大力支持民营经济发展。持续推进万名干部入企服务。精准落地面向各类企业的减税降费政策，力争全年减免各类税费45亿元以上。认真落实省市支持民营经济发展两个“30条”政策，坚决破除民营经济发展的隐性壁垒。构建“亲”“清”新型政商关系，完善领导干部联系民营企业制度，健全企业家参与涉企政策制定机制，营造支持民营企业家干事创业的良好氛围。支持成长性好的小微企业提标扩规，新增省级“专精特新”企业10户、“小升规”企业30户。继续办好“长清班”，打造素质高、眼界宽、能力强的长治优秀企业家队伍。

　　深化财税金融体制改革。强化财政资金监管，充分发挥财政评审作用，把好项目资金审核关。推进预算绩效管理体系建设，按照不低于5%的比例压减一般性支出。全面开展财源、税源调查，及时掌握新的税收增长点，加大税务稽查力度，确保应收尽收。优化金融体系结构，大力发展金融产业，积极搭建政银企合作平台，加快推进长治金融商务区建设，整合市县担保资源，组建市级融资担保集团，培育主板上市企业两家，山西股权交易中心挂牌50家，新三板挂牌两家，力争全年社会融资总量达到300亿元以上，企业资产负债率再降低1个百分点。

　　提升对外开放水平。积极融入国家“一带一路”发展战略，加强与京津冀、中原城市群、长三角、珠三角交流合作。加快对外开放平台建设，确保长治海关7月底正式开关，持续推动长治机场口岸建设，加快长子能交投口岸作业区建设。大力发展外向型经济，积极组织企业参加各类博览会，支持企业开拓国际市场，不断扩大高端煤化工、新型建材、特色农产品等优势产品出口规模，鼓励引导企业和新上项目扩大先进装备进口，新培育5家进出口潜力企业，力争出口贸易增长2%以上，实际利用外资增长3%以上。加大招商引资力度，开展系统定向精准招商，确保每个县区至少落地5个重大产业转型项目，全市招商引资签约项目当年开工率达到30%以上。

　　（四）着力乡村振兴，补齐全面小康社会短板。实施乡村振兴战略是决胜全面建成小康社会的重大历史任务。我们将坚持农业农村优先发展，统筹抓好乡村产业振兴、人才振兴、文化振兴、生态振兴、组织振兴，推动农业全面升级、农村全面进步、农民全面发展。

　　加快完善规划体系。在完成总体规划和“5+1”专项规划的基础上，按照产业带动、生态提质、城乡融合、特色保护、搬迁撤并五大发展类型，因地制宜、精准施策，分类设计好乡村建设规划。统筹生产、生活、生态三大空间，突出乡村特色，搞好风貌管控，编制实用性村庄规划，加快形成系统完备、层次分明、相互衔接的乡村规划体系。

　　大力发展特色现代农业。深入推进绿色有机旱作农业示范市和封闭示范区创建，将各县区封闭示范规模扩展到5千至1万亩。全面完成粮食生产功能区划定，加快推进沁县、屯留新增粮食产能田间工程项目，切实保障粮食安全。重点培育壮大100个农业产业化龙头企业，打造玉米、蔬菜、水果等12条农产品深加工产业链。叫响“中国小米之都”品牌，全力推进“沁州黄小米”“上党中药材”两个中国特色农产品优势区建设，打造“潞党参”“长子大青椒”等20个区域公共品牌和产品品牌，加大绿色食品、有机农产品认证力度，推进“长治神谷”建设。大力发展农村新业态，新创建200个示范合作社、家庭农场，重点扶持100个休闲农业和乡村旅游精品景点。积极培育新型职业农民和农村创业创新人才，提升农民增收致富能力。

　　持续改善农村人居环境。学习推广浙江“千村示范、万村整治”经验，以“净、绿”为抓手，实施拆违治乱、乡村绿化、垃圾治理、污水治理、厕所革命、卫生乡村六大专项行动，全面启动“百村示范、千村整治”工程，完成500个示范村绿化、200个村生活垃圾分类处理、50个村污水集中治理、9.1万座农村改厕任务。持续抓好非洲猪瘟防控工作。加大力度继续清理整治“大棚房”，坚决遏制“农地非农化”。加强传统村落保护和开发利用，让农村留得住乡韵、记得住乡愁。

　　加快推进农村综合改革。深化农村集体产权制度改革，完成清产核资和集体经济组织成员身份确认，多途径壮大村级集体经济，抓好屯留区国家级农村集体产权制度改革试点。完善农村承包地“三权分置”制度，积极推进承包地经营权抵押贷款试点工作。稳慎推动农村宅基地制度改革，开展农村宅基地使用权确权登记颁证工作。有效推动生产要素“上山下乡”，鼓励人才、政策、资金等要素向乡村流动。继续深化林权、水权制度等改革。

　　（五）着力完善基础设施，建设美丽幸福长治。基础设施建设是稳投资、扩内需、惠民生最直接最有效的举措。我们将紧紧围绕建设省域副中心城市目标定位，全力抓好城市基础设施和重大基础设施项目建设，加快推进城乡一体化发展，形成中心带动、内外联动的区域协调发展新格局。

　　狠抓城市建设。围绕拉大城市框架、完善城市功能、提升城市品质，主城区重点实施20项城建工程：1.启动主城区与上党、潞城、屯留3区的连接线城市道路标准化改造工程。2.完成207、208国道市区改线工程，208线6月底通车，207线10月底通车。3.开工建设安阳互通立交桥。4.改造长北干线、长治南高速口，畅通城市南北出口。5.开工建设高铁长治东站站房、站前广场、潞泽街等项目。6.新建高新区、职教园区周边等12条城市道路，加速产城融合发展。7.加快职教园区配套工程建设，确保6月底前全面竣工，9月投入使用。8.建成长治二十中、体育北路小学等5所学校，9月全面招生。9.全面开工博物馆、档案馆、图书馆建设。10.完善紫金西街、五针街铁路立交桥工程。11.完成环漳泽湖“五行”系统工程和滨湖大道一期工程。12.打造滨湖区起步区3平方公里景观区，为市民提供休闲健身好去处。13.改造老体育场，建设体育公园，地上健身，地下停车。14.新建15处街头游园，新增12处绿地和3处生态停车场。15.启动搬迁现有殡仪馆。16.加快长丰片区、天晚集片区棚户区改造，新开工棚户区改造3490套、基本建成6000套。17.加速北寨、小神、梁家庄等12个城中村改造。18.实施八一广场等5个片区、太行街等5条主要街道的架空线路整治。19.建成主城区生活垃圾焚烧发电厂、污泥餐厨垃圾处置项目。20.抓好“二青会”比赛场馆及周边配套设施建设，办好全国“二青会”赛事，全面展示城市美好形象。以上项目今年需要完成投资110亿元，在财力十分紧张的情况下，我们将积极争取上级资金，充分调动社会资本，市场化运作、社会化经营，确保资金落到实处，确保各项工程顺利推进。

　　狠抓基础设施建设。加快铁路、公路、机场等重大基础设施建设，构建立体联网、内外联通、多式联运的综合交通运输体系。加快推进太焦高铁建设，完成投资30亿元；开工建设总投资160亿元的黎霍高速；新建改建1000公里“四好农村路”，完成投资10亿元；抓好长治机场跑道延伸等项目，完成投资1亿元；开工建设屯留等通用机场，完成投资5亿元。做好长邯聊客专、国道208线改造、长治太行山大峡谷旅游轨道交通、壶关经陵川至云台山高速等项目的前期工作，力争早日开工建设。推动5G站址规划和基站建设。

　　统筹城乡一体化发展。立足“一城四区”大城市格局，按照“多规合一”要求，高标准修编城市总体规划，充分发挥主城区的集聚辐射功能，推动区域融合发展。持续开展违法占地、违法建设“两违”整治，有效规范房地产市场。各县区要结合自身实际，统筹规划建设一批基础设施、公共服务设施项目，全面提升县城综合承载能力。加快建立医疗卫生、劳动就业等基本公共服务跨城乡跨区域流转衔接制度，推动供热、供气、供电、供水等基础设施向农村延伸，促进基本公共服务均等化。积极推进农业转移人口市民化，全面放宽重点群体落户限制，确保常住人口和户籍人口城镇化率再提高一个百分点。

　　（六）着力文旅融合发展，打造文化旅游强市。长治是太行旅游板块的核心区域，大自然赋予了我们得天独厚的旅游资源。我们将深入挖掘、充分发挥比较优势，加快把文化旅游产业培育成战略性支柱产业，努力把长治建设成为全国一流、国际知名的全域旅游目的地。

　　加快太行旅游板块建设。在央视等知名媒体，加大宣传推介力度，叫响“山西长治·壮美太行”品牌，重点推进太行神龙湾、荫城古镇、太行霓虹水街、中太行山国际旅游度假区、常平温泉小镇等18个项目建设，完成投资13亿元。加快推进太行一号旅游公路建设，合理布局集散中心、旅游厕所、标牌标识等配套设施。开通低空旅游。扎实推进壶关、平顺、黎城、武乡4个国家全域旅游示范区创建工作。积极推进太行山大峡谷等高等级景区创建。深入实施乡村旅游扶贫重点村建设，打造30个旅游扶贫示范村。加强旅游从业人员培训，强化文旅市场综合监管，加快智慧旅游建设，全面提升旅游服务质量。

　　促进文旅深度融合发展。着力推进文化和旅游理念融合、职能融合、产业融合、市场融合、服务融合。深化景区体制机制改革，推动沁源灵空山、襄垣仙堂山等9个涉旅文物保护单位“两权分离”。推动文物活化利用，加强文创产品开发，加快推进非遗和演艺进景区。深入挖掘红色文化，积极打造旅游景区与革命遗迹相结合的红色旅游项目。大力发展文化产业，坚持创造性转化、创新性发展，打造一批文化产业基地。推进“文化+”“旅游+”，培育发展文化体验游、自驾房车游等新业态，推动文化旅游深度融合。

　　提升公共文化服务水平。积极践行社会主义核心价值观，大力弘扬太行精神。深入实施公民道德建设工程，不断提升长治文明程度和市民文明素质。强化国家公共文化服务体系示范区建设，继续办好送戏下乡、周末大剧院、农村公益电影放映、流动图书车等文化惠民活动，推动全民阅读。深入推进文明守望工程、革命文物保护利用工程和乡村文化记忆工程，加强文化遗产保护传承。繁荣文艺创作，打造文艺精品，为人民群众提供更多更好的精神食粮。

　　（七）着力三大攻坚，夯实高质量发展基础。打赢三大攻坚战是决胜全面建成小康社会的关键。我们将持续巩固扩大攻坚成果，聚焦解决突出问题，精准施策、扎实推进，打好重点战役，务求取得实效。

　　有效防范化解重大风险。防范化解重大风险是事关长远的重大政治任务，我们必须坚持底线思维，增强忧患意识，着力提高防控能力，全面排查评估研判各类重大风险和隐患，做到早识别、早发现、早预警、早处置，切实把风险化解在源头、消灭于萌芽，建立完善化解风险体制机制，综合施策、标本兼治，有效防范化解经济、社会等各领域重大风险。加大对非法金融机构和非法金融活动的打击力度，坚决防范、打击和处置非法集资，有序推进互联网金融风险专项整治。规范政府举债融资机制，遏制隐性债务增量，坚决守住不发生系统性区域性风险的底线。

　　决战决胜脱贫攻坚。今年是脱贫攻坚的决战决胜之年，要完成63个贫困村退出、2.2万贫困人口脱贫和壶关县、平顺县脱贫摘帽的攻坚任务。我们要用好用足产业扶贫专项资金，全面落实产业园区和扶贫车间奖补政策，重点打造10个特色产业示范园区，建立100个扶贫车间，惠及贫困人口3万余人。开展各类技能培训100场，培训贫困人口1万人、贫困村创业致富带头人1000人，做大做强“太行家政”特色劳务品牌，让更多贫困群众拥有一技之长。全面落实教育、健康等各项扶贫政策，确保易地扶贫搬迁人口全部入住，继续抓好农村危房改造，扎实做好“两线合一”，推动扶贫开发与农村低保政策有效衔接。坚持摘帽不摘责任、不摘政策、不摘帮扶、不摘监管，落实好已摘帽贫困县、已退出贫困村和脱贫人口的后续扶持政策，最大限度减少和防止脱贫人口返贫。加大对贫困边缘户的扶持力度，确保全面小康路上一个都不掉队。

　　坚决打好污染防治攻坚战。牢固树立绿色发展理念，强力推进生态环境保护。打赢蓝天保卫战，坚持转型、治企、减煤、控车、降尘“五管齐下”，持续开展“散乱污”企业整治，加快重污染企业搬迁改造，完成焦化行业特别排放限值改造，推进钢铁行业、燃煤机组超低排放改造；推动清洁取暖和散煤替代由城市建成区向农村扩展，完成清洁取暖改造15万户，确保所有县城建成区清洁取暖率达到100%，农村清洁取暖率达到66%；强化油品油质管控，持续开展柴油货车和散装物料运输车污染治理联合执法；严格落实扬尘治理“六个百分百”要求，确保空气质量持续改善。打好碧水保卫战，“一河一策”全面落实“河长制”，重点推进沁河、漳河流域水环境综合治理，加强辛安泉域治理保护，开工建设总长21.3公里的黑臭水体治理工程，确保主城区100%消除黑臭水体，县城及周边地区黑臭水体消除比例达到80%以上。加快城镇污水管网和污水处理厂建设，统筹推进污染严重水体和不达标水体的全流域治理，确保国考、省考水质断面稳定达标。推进净土保卫战，抓好重点行业企业用地土壤污染现状调查工作，强化农业面源污染防控，推进露天矿山综合整治，加强采煤沉陷区、矸石山治理。加强生态保护修复，大力实施太行山绿化工程，治理水土流失面积43.7万亩，创建10个森林乡镇、100个森林村庄。继续推进生活垃圾分类处理，建设完善垃圾分类处理系统，尽快形成垃圾分类投放、收集、处置的完整闭环。我们将坚决守住、守好长治的绿水青山，为全市人民提供最普惠的民生。

　　（八）着力改善民生，提升人民群众幸福指数。人民群众对美好生活的向往就是我们的奋斗目标。我们将聚焦人民群众痛点难点焦点，精心做好各项民生工作，着力解决人民群众所需所急所盼，让人民群众过上更加幸福的生活。

　　优先发展教育事业。新建改扩建农村幼儿园13所，重点扶持普惠性民办幼儿园30所，普惠性幼儿园覆盖率达到80%，学前三年毛入学率达到92%以上。推动城乡义务教育一体化发展，全面消除56人以上大班额，持续规范治理校外培训，切实减轻中小学生过重课外负担，让中小学生快乐学习、健康成长。深入实施高考质量“三年提升计划”，高考二本以上录取率进入全省前五名。优化整合职业教育资源，建立绿色农业、煤化工新能源、先进装备制造3个职业教育集团。支持和规范民办教育。强化教师队伍建设。加强校园安全风险防控和校园欺凌综合治理。

　　千方百计扩大就业。深入实施全民技能提升工程，培训5万人以上，增强劳动者就业能力。落实创业扶持政策，新增创业主体2000人，鼓励创业带动就业。扎实做好高校毕业生、去产能安置职工、农村劳动力、就业困难人员、退役军人等重点群体就业工作。鼓励企业不裁员或少裁员，稳定劳动关系。加强全方位公共就业服务，建立精准就业帮扶机制。

　　推进健康长治建设。深化县域综合医改、城市医联体建设和公立医院改革。加快医保支付方式、公立医院医药价格和药械采购三项改革，促进“三医联动”。全面完成市医院、二院、妇幼保健院改扩建工程，加快推进中医院综合楼项目，提升医疗服务能力。建设中医药强市。持续推进“双签约”“先诊疗后付费”“一站式结算”等便民惠民措施，确保贫困人口定点医疗住院综合保障比例达到90%。广泛开展全民健身活动，促进全民健身与全民健康深度融合。

　　完善社会保障制度。扎实办好省政府“八件民生实事”。继续扩大全民参保人数，认真落实城乡居民基本养老保险待遇确定和基础养老金正常调整机制，稳步提高各项社会保险待遇水平，城乡最低生活保障标准每人每月再提高30元。进一步扩大医保异地结算范围，完善城乡居民大病医保制度，把更多救命救急的新药好药纳入医保范围。提升城乡社区养老服务水平，社区养老中心覆盖率达到70%以上。深入开展农村特殊群体关爱、残孤儿童生活保障工作，加强和改进流浪乞讨人员救助管理，大力发展妇女、儿童、老龄、慈善、残疾人和红十字等事业。

　　（九）着力安全稳定，提高社会治理水平。安全重于泰山，稳定压倒一切。我们将全面贯彻总体国家安全观，牢牢守住社会安全稳定这一底线，确保人民安居乐业、社会安定有序。

　　切实抓好安全生产。严格落实党政领导干部责任制规定，压实部门监管责任，严格按照“管行业必须管安全、管业务必须管安全、管生产经营必须管安全”和“谁主管谁负责”的要求，构建形成政府和所有负有安全监管职责的部门“1+X”清单管理体系。严格落实企业主体责任，做到安全责任、管理、投入、培训和应急救援“五到位”。持续深化煤矿、非煤矿山、危化品、建筑施工、燃气、特种设备等重点行业领域专项整治，夯实基层基础，坚决杜绝重特大事故，确保全市安全生产形势稳定。

　　深化平安长治建设。进一步深化扫黑除恶专项斗争，持续开展禁毒三年攻坚战，严厉打击各类违法犯罪活动，努力打造全省最安全城市。加强网络安全建设。提高风险预知预警预判能力，完善应急预案体系，强化应急救援队伍建设，不断增强应急保障和处置能力，严防群体性事件、突发环境事件、重大食品药品安全事故、重大交通事故等公共安全事件。加强气象、地震、人防等工作，做好重大自然灾害的防灾减灾救灾工作。

　　加强社会综合治理。建设信用长治、诚信长治，提升全社会诚信水平。学习推广“枫桥经验”，全面加强基层社会治理。完善基层群众自治制度，加强社区治理，更好发挥工会、共青团、妇联等群团组织作用，推动社会治理重心向基层下移。完善社会矛盾纠纷多元化解机制，创新信访工作方式，做实做细各类利益诉求群体的信访维稳工作。支持国防建设，做好退役军人服务管理和双拥工作。加强民族宗教、外事侨务和援疆工作。做好统计、档案、地方志等各项工作。

　　各位代表，新时代新使命对政府自身建设提出了更高要求。省委作出开展“改革创新、奋发有为”大讨论的重大决策部署，我们将以此为契机，进一步提高政治站位，结合实际组织开展好大讨论，不断增强“四个意识”、坚定“四个自信”，坚决做到“两个维护”，围绕“六个破除”“六个着力”“六个坚持”，聚焦思想再解放、改革再深入、创新再发力、开放再提质、工作再抓实，在全市政府系统真正来一场学习的革命、思想的革命、工作的革命，推动理念大提升、本领大提升、作风大提升，汇聚起攻坚克难、勇往直前的强大力量，不断提高政府治理能力现代化水平。

　　一要严格依法行政。建设法治政府是实现政府治理能力现代化的关键。各级政府将坚持依宪施政、依法行政，严格按照法定权限和法定程序行使权力，自觉运用法治思维和法治方式推进工作。坚持依法决策，健全完善程序，充分发扬民主，对重大民生政策出台、重大公共项目建设要依法公示听证，切实提高行政决策的科学化、民主化、法治化水平。全面完成市县两级政府机构改革。推进综合行政执法体制改革，严格规范公正文明执法。严格执行人大及其常委会的决议决定，认真办理人大代表建议、政协提案，自觉接受人大、政协监督，主动接受社会、舆论和网络监督。

　　二要勇于担当作为。新的征途不会平坦，前进道路充满挑战。我们将以对发展负责、对历史负责、对人民负责的态度，始终保持锐意进取、永不懈怠的精神状态和敢闯敢干、一往无前的奋斗姿态，敢于直面矛盾、迎难而上，勇于挑最重的担子、啃最硬的骨头，善于想办法、出实招破解经济社会发展难题，真正做到守土有责、守土负责、守土尽责。深化“13710”督办制度，确保政策落地、政令畅通。健全容错纠错机制，为担当者担当、为负责者负责、为干事者撑腰，营造担当作为的良好氛围。

　　三要创优发展环境。权力来自人民必须服务人民。各级政府将牢固树立以人民为中心的思想，坚持服务至上，围绕打造“六最”营商环境，深化“放管服效”改革。持续引深审批制度改革，推行“承诺制+并联审批”模式，推进“承诺制+标准地”改革，巩固深化“3545”专项改革，大幅压缩企业开办、不动产登记、一般性工业项目审批时间，力争全年新登记市场主体数量增长10%。不断创新监管方式，推进“双随机、一公开”监管统一化、常态化，推动跨部门联合检查，规范涉审中介组织行为，大力整治“红顶中介”。加快推进“互联网+政务服务”，深化“一门一网一次”改革，在市县两级政务服务中心全面推行“前台综合受理、后台分类审批、综合窗口出件”工作模式，让更多事项“一网通办”，实现“只进一扇门、最多跑一次”，最大限度提升企业和群众办事便利度和满意率。

　　四要保持清正廉洁。为政之要在于廉洁。我们将严格落实政府系统全面从严治党主体责任，深入推进政府系统党风廉政建设和反腐败斗争。坚决贯彻落实中央八项规定精神，坚决反对形式主义、官僚主义，驰而不息整治“四风”。切实加强审计监督。全面推进政务公开和信息公开，让权力在阳光下运行。全体公务人员都必须牢固树立“人人代表政府形象”理念，始终严格要求自己，自觉遵循纪律规矩，永葆为民务实清廉的政治本色。

　　各位代表，长治的历史灿烂辉煌，长治的未来充满希望。前进征程中面临许多风险和挑战，我们将披荆斩棘、勇往直前。让我们高举习近平新时代中国特色社会主义思想伟大旗帜，在省委、省政府和市委的坚强领导下，不忘初心、牢记使命，改革创新、奋发有为，全力打造山西重要增长极、建设省域副中心城市、建设美丽幸福长治，以优异成绩迎接新中国成立70周年！
